# Sonic Hedgehog reduces inflammatory response, decreases blood-spinal cord barrier permeability, and improves locomotor function recovery in an acute spinal cord injury rat model

**DOI:** 10.1186/s12950-024-00404-y

**Published:** 2024-09-03

**Authors:** Mohamed Tail, Hao Zhang, Guoli Zheng, Anna-Kathrin Harms, Maryam Hatami, Thomas Skutella, Karl Kiening, Andreas Unterberg, Klaus Zweckberger, Alexander Younsi

**Affiliations:** 1 Department of Neurosurgery, Heidelberg University Hospital, 69120 Heidelberg, Germany; 2 Department of Neurology, Heidelberg University Hospital, 69120 Heidelberg, Germany; 3 Department of Neuroanatomy, Institute for Anatomy and Cell Biology, Heidelberg University, 69120 Heidelberg, Germany

**Keywords:** SCI, BSCB, Spinal cord injury, Sonic Hedgehog, Inflammation

## Abstract

**Background:**

Sonic Hedgehog (Shh), extensively researched for its role in early neurogenesis and brain development, has recently been recognized for its neuroprotective potential following neuronal injuries. This study examines the immediate impact of early administered Shh on the local inflammatory response post-acute spinal cord injury in rats.

**Methods:**

Thirty-four female Wistar rats underwent either sham surgery (laminectomy; *n* = 10) or clip compression/contusion spinal cord injury (SCI) at the T9 level. This was followed by implantation of an osmotic pump and a subdural catheter for continuous intrathecal delivery of Shh (*n* = 12) or placebo (NaCl; *n* = 12). Locomotor function was assessed at 3- and 7-days post-injury (dpi) using the Basso, Beattie, and Bresnahan (BBB) score and the Gridwalk test. Animals were euthanized after 3 or 7 days for immunohistochemical analysis of the local inflammatory reaction and immune cell migration.

**Results:**

Shh-treated rats demonstrated significant hindlimb movement and coordination improvements at 7 days post-injury, compared to controls. This enhancement was accompanied by a significant reduction in both immune cell presence and blood plasma products within spinal cord lesions, suggesting Shh’s dual role in modulating immune cell migration and maintaining the integrity of the blood-spinal cord barrier. Separately, these Shh-treated rats also showed an increase in M(IL-4) polarization of macrophages, further underlining the potential therapeutic impact of Shh in post-injury recovery. Notably, these effects were not evident at three days post-injury.

**Conclusion:**

Shh application at 7 days post-injury showed immunomodulatory effects, possibly via enhanced blood-spinal cord barrier integrity, reduced immune cell migration, and increased anti-inflammatory immune cell differentiation. These mechanisms collectively contribute to enhanced locomotor recovery.

## Background

Decades of spinal cord injury (SCI) research yielded a substantial understanding of the pathophysiological processes associated with this devastating injury but also of potential targets for neuroprotective or regenerative therapies. While no feasible treatment has surfaced out of these long-lasting efforts yet, recent publications still show ongoing innovation and new ideas in the fight against neurological impairment after SCI [1, 2]. As such, Sonic Hedgehog (Shh) has been identified as a potential player that could be used for SCI treatment: In preclinical studies, Shh appeared to have neuroprotective and neuroregenerative effects on the injured spinal cord and its proliferative effect on young neurons has even shown to be an adjuvant component when applied in stem-cell-based therapies [3, 4].

Inflammation and scarring, as part of the body’s repair mechanisms, are needed to mitigate further trauma and separate damaged tissue from healthy tissue. However, in neurological tissue, post-injury inflammatory processes do not only exacerbate current damage rather than mitigate it, but they undermine the potential of regeneration by inducing scarring with loss of function [1, 5–7]. In the initial acute (< 24 h) and subacute (24 h-7d) phases after SCI, mechanical damage sets off a cascade of post-injury inflammation processes that, in general, tend to worsen already acquired neurological deficits [5, 7]. Furthermore, patients with SCI show signs of chronic lesion site inflammation, perpetuating not only further damage but also encumbering long-term compensating mechanisms to be channeled [8, 9].

In previous works, we were able to measure long-term inflammation attenuating effects of Shh after SCI when applied one week after the injury [4]. This study aims to understand the potential impact of Shh on immediate secondary injury cascades and its role in early repair mechanisms.

## Materials and methods

Animals, Treatment Characteristics, and Study Design.

We used 34 female Wistar rats (four weeks old, 160 g; *Janvier Labs*,* France*) in three treatment groups at two different timepoints (3- and 7-days post injury (dpi)): Group 1 (Shh; 3 dpi: *n* = 6; 7 dpi: *n* = 6), group 2 (Control; 3 dpi: *n* = 6; 7 dpi: *n* = 6) and group 3 (Sham; 3 dpi: *n* = 5; 7 dpi: *n* = 5). Rats were housed in 1815 cm^2^ cages and food and water ad libitum, a 12-h light-dark cycle, and temperature at 26° were maintained. No possibility for self-training was given. Weighing of the animals was performed daily. Neurological function was assessed at baseline at 3 dpi and at 7 dpi by two independent observers. For intrathecal administration of Shh or placebo we used osmotic micropumps (*Alzet*,* USA*) implanted immediately after SCI. Two animals in group 1 died during the experiment, resulting in a total sample size of *n* = 32 rats. The study was terminated with the perfusion of all animals at respective time points (3 dpi, 7 dpi). All experimental protocols were approved by the Animal Care Committee of the federal government.

### Surgical interventions

We described the performed surgical interventions in our previous work [10]. In short, animals were put under general anesthesia with isoflurane (1.5–3%) and a 1:1 mixture of O_2_ and N_2_O. Laminectomy was performed at the T9 level. After exposing the spinal cord, we reproduced a contusion–compression injury using a 28 g modified aneurysm clip (*Fehlings Laboratory*,* Canada*). We applied it around the spinal cord, shut and sustained it for 60 s. To administer Shh or placebo to the spinal cord, we implanted a subcutaneous osmotic pump (*model 1007D; Alzet*,* USA*) right after SCI and placed the tip of the connected rat microcatheter subdurally via a skip laminectomy of T11 and fixation to the paraspinal muscles. Osmotic pumps were preloaded with 100 µl of Shh (50 ng/ml; *R&D Systems*,* USA*; group 1) or 0.9% NaCl (group 2; Control), primed in a 2-hour loading cycle in 0.9% NaCl at room temperature and randomly assigned to the animals intraoperatively. After implantation, the osmotic pumps delivered the loaded substances directly to the epicenter of the lesion for up to seven days or until the time of sacrifice. Sham animals (group 3) received a laminectomy of T9 and T11 only. The postoperative protocol included antibiotic prophylaxis using 4 mg/kg moxifloxacin p.o. (*Fresenius*,* Germany*) analgesia using 0.05 mg/kg buprenorphine s.c. (*Bayer*,* Germany*) and 2 mg/kg meloxicam s.c. (*Boehringer-Ingelheim*,* Germany*), as well as fluid substitution (3–5 ml 0.9% NaCl s.c.) for up to 5 days. Bladders were manually voided twice daily until reflexive bladder function had recovered.

### Assessment of locomotor function

To objectively assess hindlimb movement we performed an open-field test and evaluated it according to the Basso-Beattie-Bresnahan locomotor rating scale (BBB) at 1, 3 and 7 dpi. Points were given for hindlimb movement, weight support, coordination, joint involvement, tail movement, and trunk position in a four-minute run, evaluated by two blinded observers (Table 1). Results were averaged and statistically analyzed (0–21 points) [11].

**Table 1:** To evaluate post-SCI locomotor function, the Basso, Beattie, Bresnahan (BBB) score for rodents was used [11]. Animals were tested at 1, 3 and 7 dpi. Two blinded observers scored each rat in an open field box

Score	Description
0	No observable hindlimb (HL) movement
1	Slight movement of one or two joints, usually the hip and/or knee
2	Extensive movement of one joint or extensive movement of one joint and slight movement of one other joint
3	Extensive movement of two joints
4	Slight movement of all three joints of the HL
5	Slight movement of two joints and extensive movement of the third
6	Extensive movement of two joints and slight movement of the third
7	Extensive movement of all three joints of the HL
8	Sweeping with no weight support or plantar placement of the paw with no weight support
9	Plantar placement of the paw with weight support in stance only (i.e., when stationary) or occasional, frequent, or consistent weight supported dorsal stepping and no plantar stepping
10	Occasional weight supported plantar steps, no forelimb (FL)-HL coordination
11	Frequent to consistent weight supported plantar steps and no FL-HL coordination
12	Frequent to consistent weight supported plantar steps and occasional FL-HL coordination
13	Frequent to consistent weight supported plantar steps and frequent FL-HL coordination
14	Consistent weight supported plantar steps, consistent FL-HL coordination; and predominant paw position during locomotion is rotated (internally or externally) when it makes initial contact with the surface as well as just before it is lifted off at the end of stance or frequent plantar stepping, consistent FL-HL coordination, and occasional dorsal stepping
15	Consistent plantar stepping and consistent FL-HL coordination; and no toe clearance or occasional toe clearance during forward limb advancement; predominant paw position is parallel to the body at initial contact
16	Consistent plantar stepping and consistent FL-HL coordination during gait; and toe clearance occurs frequently during forward limb advancement; predominant paw position is parallel at initial contact and rotated at lift off
17	Consistent plantar stepping and consistent FL-HL coordination during gait; and toe clearance occurs frequently during forward limb advancement; predominant paw position is parallel at initial contact and lift off
18	Consistent plantar stepping and consistent FL-HL coordination during gait; and toe clearance occurs consistently during forward limb advancement; predominant paw position is parallel at initial contact and rotated at lift off
19	Consistent plantar stepping and consistent FL-HL coordination during gait; and toe clearance occurs consistently during forward limb advancement; predominant paw position is parallel at initial contact and lift off; and tail is down part or all of the time
20	Consistent plantar stepping and consistent coordinated gait; consistent toe clearance; predominant paw position is parallel at initial contact and lift off; tail consistently up; and trunk instability
21	Consistent plantar stepping and coordinated gait, consistent toe clearance, predominant paw position is parallel throughout stance, consistent trunk stability, tail consistently up

Furthermore, the Gridwalk test was performed on a 1 m long runway of evenly spaced metal bars with bars randomly missing. Stepping errors were counted by two blinded observers for every misplaced step per hindlimb and averaged over three trials. Baseline training and evaluation were performed one day prior to injury, at 3 dpi and 7 dpi [12].

### Immunofluorescence staining and imaging analysis

After animal sacrifice and perfusion with cold phosphate buffered saline (PBS) and 4% paraformaldehyde (PFA), as previously described [4, 13] we obtained spinal cord pieces +/- 10 mm from the epicenter which were incubated with 4% PFA for 24 h and 30% sucrose for 48 h. The spinal cord pieces were then embedded with Tissue-TEK^®^ (*Sakura Finetek Europa B.V.*,* Netherlands*) on dry-ice and cut into consecutive 30 μm thick cross-sections using a cryostat (*Leica Biosystems*,* Germany*). Finally, spinal cord cross-sections were placed on specimen slides, dried, and stored at -80 °C until further usage.

For immunofluorescence staining, slides were thawed at room temperature and washed three times with PBS. The cross-sections were then subjected to the following immunofluorescence staining protocol: PBS was removed, and a blocking solution containing 0.3% Triton-X100, 5% milk powder, and 1% bovine serum albumin (all *Sigma-Aldrich*,* USA*) was added for one hour at room temperature. Then, the cross-sections were incubated with primary antibodies diluted in the blocking solution at 4° C overnight.

The following primary antibodies were used: Anti- β-catenin (1:200; rabbit; *Abcam*,* USA*) for the evaluation of adherens junction complexes, anti-Fibrinogen (1:500; mouse; *Santa Cruz*,* Germany*) for blood plasma protein leakage, anti-TMEM119 (1:200; rabbit; *Abcam*,* USA*) as a marker for resident microglia, anti-Iba1 (1:200; goat; *Novus Biologicals*, USA) for macrophages and microglia, anti-CD206 (1:200; goat; *Bio-Rad*,* Germany*) and anti-iNOS (1:100; mouse; *Abcam*,* USA*) for the identification of M(IFN-γ)- and M(IL-4)- macrophages.

Next, we washed the cross-sections three times with PBS and subjected them to incubation with the secondary antibodies diluted in a blocking solution without Triton-X100 for one hour at room temperature. The following secondary antibodies were used: Alexa Fluor 557 donkey anti-mouse (1:400; *R&D Systems*,* USA*), Alexa Fluor 647 donkey anti-rabbit (*1:400; Abcam*,* USA*), and Alexa Fluor 405 donkey anti-goat (1:400; *Abcam*,* USA*).

Using a confocal laser scanning microscope (*LSM 700; Carl-Zeiss*,* Germany*) we obtained images of each cross-section at 10x magnification in an 8-bit-format with tile scan function (speed of 4, gain of 800). Four wavelength channels (Alexa Fluor-405 nm, GFP-488 nm, Alexa Fluor 568 nm, Alexa Fluor 647 nm) were used.

To quantitatively evaluate the different cell types on the cross-sections, we applied a semi-automatic counting-algorithm for ImageJ2 (National Institute of Health, Bethesda, USA), as previously described [4]. In short, images were split into single channels using the ImageJ2 software [14]. Then, a region of interest (ROI) was placed, covering the whole cross-sectioned spinal cord. Following transformation into a binary image using the “IsoData-threshold” function, a binary image was formed and the “Analyze” function was used to determine the cell count. To quantify co-labeled cells within the selected ROI, we used the “Image calculator” function to combine channels before transformation into binary images. To avoid the inclusion of artifacts, only cells with an area of 50-2000 µm^2^ were considered. Cell counts where then divided by the area of the ROI (derived with the “Measure” function), and the results of 40 cross-sections per animal (+/- 600 μm from the lesion epicenter) were averaged and expressed as cells/mm^2^.

To quantitatively evaluate BSCB leakage (ß-Catenin and Fibrinogen), the immunointensity of the respective antibody fluorescence was quantified, as previously described [4, 15]. Briefly, we split images into single channels with the ImageJ2 software, set ROIs covering the whole cross-sectioned spinal cord, and used the “Measure” function to derive the area and the integrated density in pixels (pi). Integrated density values were then divided by the area of the ROI, the results of 40 cross-sections per animal (+/- 600 μm from the lesion epicenter) were averaged and expressed as pi/mm^2^.

### Statistical analysis

We performed Shapiro-Wilk normality tests prior to normality assumption before all parametric analyses. One-way analysis of variance (ANOVA) followed by post hoc Tukey-HSD tests were used for the statistical comparison of means between multiple groups. Two-way ANOVA followed by post hoc Tukey-HSD tests were performed for the statistical comparison of means between multiple groups among multiple time points. For the comparison of two groups, unpaired student t-tests were performed. All results are given as mean ± standard error of the mean (SEM), and *p* < 0.05 was considered significant. All statistical analyses were done with the software Prism (*GraphPad Software*,* USA*) in version 9.

## Results

### Shh-treatment might decrease BSCB permeability

Fibrinogen is a blood serum protein that, under physiological circumstances, cannot pass the BSCB. To understand Shh-mediated effects on BSCB permeability, we examined the intramedullary concentration of Fibrinogen after SCI as a surrogate marker for perivascular leakage. While there was no significant difference between the untreated and Shh-treated animals at 3 dpi (12.51 ± 0.9468 pi/mm^2^ vs. 16.09 ± 1.846 pi/mm^2^; *p* = 0.1721), we observed significantly less intramedullary Fibrinogen at 7 dpi with the Shh-treatment (16.16 ± 1.208 pi/mm^2^ vs. 26.03 ± 1.192 pi/mm^2^; *p* < 0.01). These results might indicate a Shh-related decrease of the post-traumatic BSCB permeability.

The highly regulated permeability of the BSCB is largely dependent on cellular adhesion complexes. An Shh-associated increase of tight junction proteins has been well documented. Next to the Shh-pathway, the Wnt/β-Catenin pathway has been deemed important for maintenance and repair of BBB [16–18].

We chose to examine the expression of β-Catenin, an adherens junction and crucial Wnt/β-Catenin-pathway protein, to assess possible Shh-mediated alterations.

**Fig. 1:**
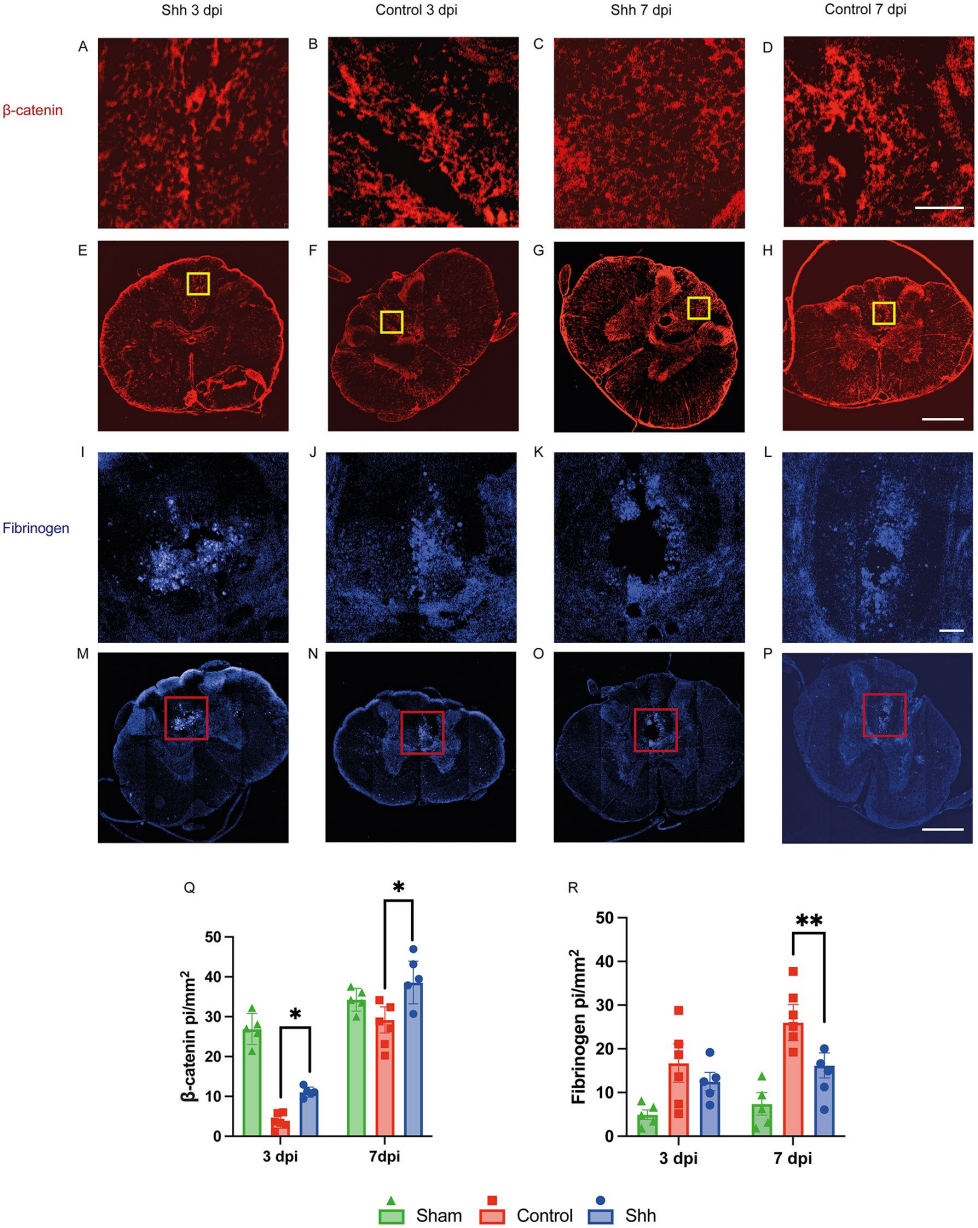
(**A-P**) Cross-sectional images of lesioned spinal cords in the Shh- and Control group 3- and 7-days post injury (dpi), stained for β-catenin (red) and Fibrinogen (blue) at different magnifications: 20x (**A-D**; scale bar: 150 μm), 10x (I-L; scale bar: 75 μm), and 10 × (4x4 tile-scans, E-H + M-P; scale bar: 500 μm). (**Q**) β-catenin staining (red) shows a significantly higher concentration in Shh-treated animals (*n* = 5) compared to untreated animals at 7 dpi (*p* = 0.0234, *n* = 6), as well as at 3 dpi (*p* = 0.0213). Blue staining highlights intramedullary evidence of Fibrinogen extravasation (I-P; blue). (**R**) While there was no significant difference in Fibrinogen accumulation between Shh-treated (*n* = 5) and untreated animals (*n* = 6) at 3 dpi, at 7 dpi, less Fibrinogen extravasation was observed in the Shh group (*n* = 5) compared to the Control group (*p* < 0.01, *n* = 6). Data are expressed as mean ± SEM pi/mm². Statistical analysis was conducted using one-way ANOVA with post hoc Tukey-HSD tests (*p < 0.05, **p < 0.01)

Interestingly, Shh treated animals showed significantly higher β-Catenin levels compared to untreated animals at 3 dpi (11.06 ± 0.749 pi/mm^2^ vs. 3.869 ± 0.910 pi/mm^2^; *p* = 0.0213) as well as at 7 dpi (38.59 ± 3.386 pi/mm^2^ vs. 29.36 ± 2.015 pi/mm^2^; *p* = 0.0234) (Fig. 1).

These results may implicate a Shh-mediated modulation of β-Catenin concentration and consecutively interaction with the Wnt/β-Catenin pathway.

### Shh-pathway activation might reduce trans-BSCB migration of blood-borne immune cells

Recent publications attribute an anti-inflammatory effect to the Shh-pathway activation in neuronal settings [19–22]. The underlying mechanisms of these effects are not fully understood. However, the extent of neuro-inflammation is associated with the amount of pro-inflammatory proteins and cells present which in turn might be related to trans-BSCB migration [23, 24]. We thus semiautomatically quantified macrophages in the spinal cord expressing Iba-1 in the absence of TMEM119, indicating a blood-borne origin and potential trans-BSCB migration.

**Fig. 2:**
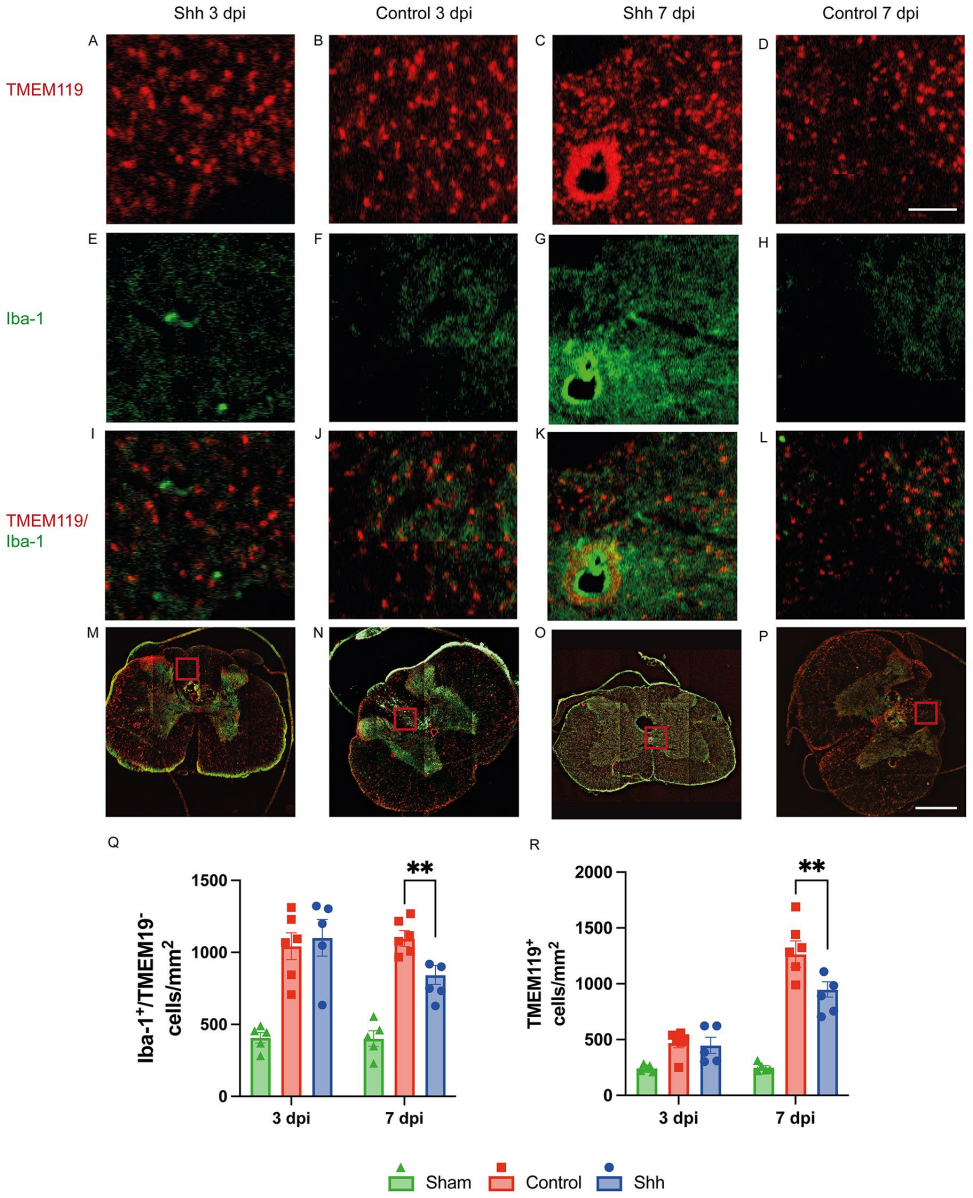
(**A-L**) 20x-magnification cross-sectional images of lesioned spinal cords in the Shh- and Control group 3- and 7-days post injury (dpi) stained for TMEM119 (red; **A-D**) and Iba-1 (green; **E-H**), along with composite images (**I-L**; scale bar: 150 μm). (M-P) The corresponding 10x-magnification 4 × 4 tile-scans are presented as composite images, with the overlying inlets (**A-L**) depicted in red (scale bar: 500 μm). (**Q**) TMEM119 is expressed by intramedullary resident microglia, while blood-borne immune cells lack TMEM119 expression. Therefore, subtracting TMEM119^+^ cells from the total Iba-1^+^ macrophage population enables the identification of infiltrating macrophages. Shh-treated animals (*n* = 5) exhibited a significantly lower number of Iba-1^+^/TMEM119^−^ infiltrating immune cells compared to untreated controls (*n* = 6) at 7 dpi (*p* = 0.0027). (**R**) Additionally, fewer TMEM119^+^ resident microglia were observed in the Shh group (*n* = 5) compared to the Control group (*n* = 6) at 7 dpi (*p* < 0.01). Data are presented as mean ± SEM cells/mm². Statistical analysis was performed using one-way ANOVA with post hoc Tukey-HSD tests (**p < 0.01)

At 7 dpi, the presence of such Iba-1^+^/TMEM119^−^ cells in the injured spinal cord was significantly lower in Shh-treated animals than in untreated animals (838.54 ± 43.57 cells/mm^2^ vs. 1093.54 ± 48.87 cells/mm^2^; *p* = 0.0027), which might be attributed to Shh-related tightening of the BSCB. This effect could not be seen at 3 dpi, where Shh-treated and Control animals did not significantly differ in the number of infiltrated cells (1091.45 ± 96.46 cells/mm^2^ vs. 1043.38 ± 61.40 cells/mm^2^; *p* = 0.837). Furthermore, at 7 dpi, Shh-treated animals also displayed less intramedullary TMEM119^+^ cells compared to untreated animals in the Control group (954.13 ± 60.14 cells/mm^2^ vs. 1262.78 ± 60.66 cells/mm^2^; *p* < 0.0001), while at 3 dpi no significant difference was found (417.84 ± 68.27 cells/mm^2^ vs. 483.30 ± 45.50 cells/mm^2^; *p* = 0.829). Since the expression of TMEM119 characterizes resident microglia, this finding might be interpreted as a reduced immune cell activation under the Shh pathway activation (Fig. 2).

### Shh is related to anti-inflammatory polarization of macrophages

The post-injury secondary inflammatory changes in the spinal cord are partly defined by the prevalent ratio of immune cells in situ [25]. A transition from pro-inflammatory to anti-inflammatory macrophages is thereby associated with less neuronal damage and improved locomotor function rehabilitation after SCI [25–27]. To determine the polarization of macrophages in the injured spinal cord, we used the markers iNOS for the M(IFN-γ)- and CD206 for the M(IL-4)-phenotype and semiautomatically counted cells with either only iNOS- or CD206-expression, thereby disregarding macrophages still in transition (expressing both, iNOS and CD206).

**Fig. 3:**
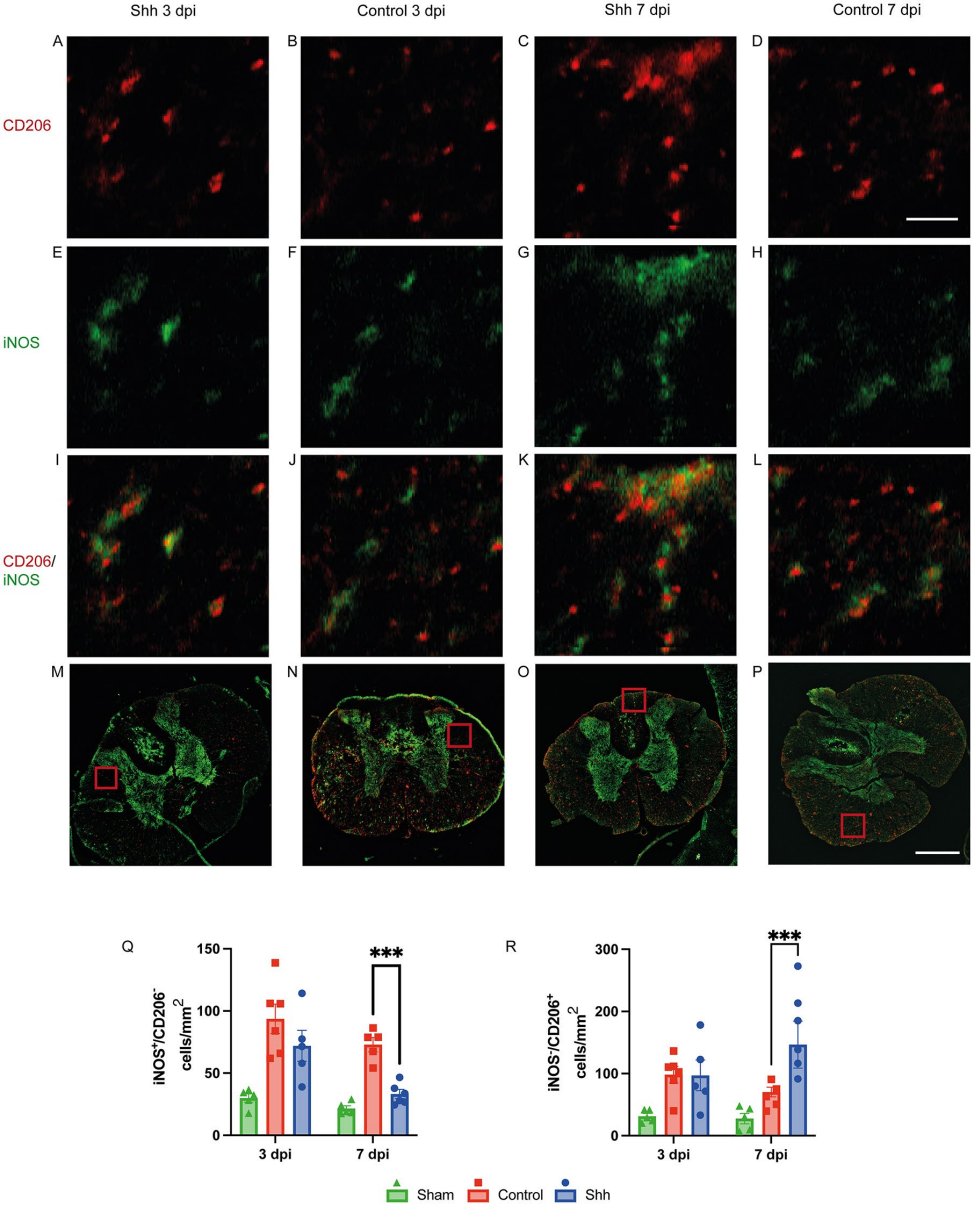
(**A-L**) 20x-magnification cross-sectional images of lesioned spinal cords in the Shh- and Control group 3- and 7-days post injury (dpi) stained for CD206 (red; A-D), iNOS (green; E-H), and composite images (I-L; scale bar: 150 μm). (M-P) The corresponding 10x-magnification 4 × 4 tile-scans are presented as composite images, with the overlying inlets (**A-L**) depicted in red (scale bar: 500 μm). Due to the fluid nature of M(IFN-γ) to M(IL-4) polarization, distinguishing between the expression of CD206 and iNOS remains challenging. Therefore, iNOS-expressing cells without CD206 co-staining (iNOS^+^/CD206^−^) were counted as indicative of M(IFN-γ)-polarized macrophages, while iNOS^−^/CD206^+^ cells were considered indicative of M(IL-4)-polarized macrophages. (Q) While no significant difference was observed at 3 dpi, Shh treatment (*n* = 5) was associated with significantly fewer M(IFN-γ)-polarized, pro-inflammatory macrophages compared to untreated controls at 7 dpi (*p* < 0.001, *n* = 6). (R) Simultaneously, an increase in M(IL-4)-polarization towards an anti-inflammatory macrophage phenotype was noted under Shh treatment (*n* = 5) compared to untreated animals (*p* < 0.001, *n* = 6). Data are expressed as mean ± SEM cells/mm². Statistical analysis was performed using one-way ANOVA with post hoc Tukey-HSD tests (***p < 0.001)

At 3 dpi we could not find a difference between Shh-treated and Control animals in neither the number of iNOS^+^/CD206^−^ M(IFN-γ)-polarized macrophages (M(IFN-γ)) (71.97 ± 12.45 cells/mm^2^ vs. 93.01 ± 15.33 cells/mm^2^; *p* = 0.168), nor iNOS^−^/CD206^+^ M(IL-4)-polarized macrophages (M(IL-4)) (94.32 ± 20.32 cells/mm^2^ vs. 98.71 ± 11.49 cells/mm^2^; *p* = 0,973) in situ. However, the Shh-treatment led to a significantly lower number of M(IFN-γ) compared to the Control group 7 dpi (32.85 ± 2.54 cells/mm^2^ vs. 72.66 ± 6.99 cells/mm^2^; *p* < 0.001). Furthermore, Shh-treated animals showed a higher count of M(IL-4) than the untreated Control animals (150.78 ± 24.93 cells/mm^2^ vs. 70.65 ± 10.37 cells/mm^2^; p = *p* < 0.001), indicating that Shh might be related to an increased transition from the pro-inflammatory M(IFN-γ)- to the anti-inflammatory M(IL-4)-phenotype (Fig. 3).

### Shh-application is associated with improved functional recovery of hindlimb locomotion

**Fig. 4:**
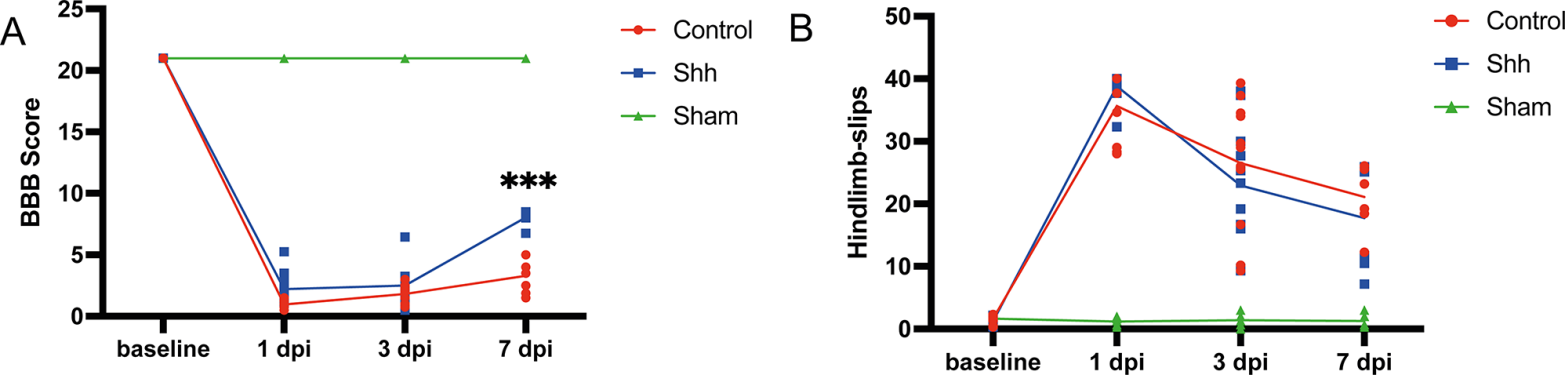
(**A**) Shh-treated animals (*n* = 5) demonstrated improved locomotor function, as indicated by significantly higher BBB scores at 7 days post-injury (dpi) compared to untreated control animals (*p* < 0.001, *n* = 6). (**B**) In the Gridwalk test, both the Shh- and the control group showed a similar rate of stepping errors at 3 dpi and 7 dpi (for both timepoints: Shh: *n* = 5, Control: *n* = 6). Experiments were conducted in triplicate, with data interpreted by two blinded observers and averaged. Data are expressed as BBB points or stepping errors and presented as mean ± SEM. Statistical analysis was performed using two-way ANOVA with post hoc Tukey-HSD tests (***p < 0.001)

In order to examine acute effects off Shh on the functional outcome after SCI we tested for hindlimb locomotion using the Gridwalk test and the BBB locomotor rating scale. The Gridwalk test did not display significant differences between the Shh- and the Control-group at 3 dpi (23.07 ± 2.64 stepping errors vs. 25.89 ± 2.61 stepping errors; *p* = 0.613) and 7 dpi (18.10 ± 2.75 vs. 20.67 ± 2.63 stepping errors; *p* = 0.643). The evaluation of the BBB scores at 3 dpi showed no significant difference between both group (Shh 2.47 ± 0.42 points vs. Control 1.69 ± 0.170 points; *p* = 0.183). However, at 7 dpi, a significant and relevant improvement of hindlimb locomotor function in Shh-treated animals compared to untreated Control animals could be observed (7.857 ± 1.262 points vs. 3.583 ± 1.175 points; *p* < 0.001, Fig. 4).

## Discussion

SCI is a devastating diagnosis for patients and involved relatives. Years of neuroregenerative research advanced our understanding of pathophysiological processes but failed to find a feasible treatment to rehabilitate patients. Promising substances and treatments have previously been advanced into phase II/III trials [1]; however, none of these have yielded relevant results yet [28, 29].

It is imperative to understand that treatment begins with prevention. For example, improved vehicle and work safety measures have shown a steady decrease in traumatic SCI in Europe and the US. After initial injury, further therapeutic options are scarce. We believe that breaking the cycle of secondary injury after SCI as prevention of further damage might be a treatment option itself, leading to improved functional outcomes and life quality.

Shh and the Shh-pathway have been extensively researched on their morphogenic as well as proto-oncological capability [30–32]. Recent publications introduced the neuroprotective and anti-inflammatory features of Shh on neuronal tissue. Furthermore, its endogenous activation after neuronal injury [3, 21], as well as its effect on the recruitment and guidance of undifferentiated neuronal cells has been associated with the active repair of neuronal damage [33].

Therefore, through our experimental study design, we aimed to understand Shh-pathway-associated repair and defensive mechanisms in the acute and early subacute SCI environment. Previous studies examining intrathecal substance-application had varying timepoints of insertion ranging from 3 days pre-injury to 7 days post-injury [34–36]. Since our aim was to study and understand Shh-mediated changes in the acute and subacute phase, our approach to implant the intrathecal pump at the same time as SCI seemed a sensible approach. Like other researchers, we suspect that crucial effects of treatment might take place early, since the BSCB is most vulnerable in the acute stages of injury [37, 38].

Interestingly, we were able to show that Shh-treated rats exhibit significantly less plasma protein leakage after injury compared to untreated animals. This is in accordance with prior findings. For example, Alvarez et al. 2011 described increased Fibrinogen extravasation in Cyclopamine injected mice, a potent Shh-pathway inhibitor [39]. The underlying mechanisms are still unknown. Different effector proteins have been identified as being induced by GLI-1, one of the main transcriptors activated by the Shh-pathway, e.g., Netrin-1 and β-catenin [40, 41].

In our study, we could depict a significant increase in β-catenin concentration in Shh-treated animals. The dual function of β-catenin not only serves as a signal transducer - essential to the Wnt/β-catenin pathway - but also as a vital adherents junction protein to the blood-brain-barrier complex [42]. In review of known data, the effects of the Wnt/β-catenin-pathway go beyond tight junction modulation to increase the BBB. Induction of BBB properties in endothelial cells, regulation of transcytosis as well as reduction of inflammatory responses are processes by which the pathway might enhance the BBB. Like Shh-pathway activation, Wnt-/β-catenin pathway activation is associated with increased expression of tight junction proteins such as ZO-1, Occlodin and Claudin 5. [43–45]. Changes in the perivascular concentration of β-catenin are associated with changes in BSCB permeability, and as such they are commonly used as an indicator for BBB and BSCB disruption [46]. The Shh-related increase in β-catenin might suggest a synergistic effort on BSCB tightening attenuation. [47]

The disruption of the BSCB in trauma is considered part of the posttraumatic inflammation process, as it facilitates an influx of immune cells and other blood-derived immune factors to further the immune response on site [42, 48, 49]. This might be the key reason for our observed decreased migration of recruited macrophages into the lesion site. In previous experiments we were able to show attenuated neuroinflammation via Shh-pathway activation in vitro and in vivo [4, 15]. One key mechanism might be the described BSCB tightening that disallows immune cell migration. Albeit the association between BSCB and immune cell immigration might seem self-evident, it has not been thoroughly elaborated yet and will require further investigation.

While pro-inflammatory M(IFN-γ)-macrophages are associated with IL-12, IL-23, and tumor necrosis factor-α (TNF-α) expression and help “irrigate” the lesion site, M(IL-4)-macrophages are characterized inter alia by CD206 surface proteins and show secretion of anti-inflammatory interleukins and chemokines (IL-10, CCL17, CCL18, and CCL22) to counter the inflammatory response and start the wound healing phase [50–53]. Extensive research on M(IFN-γ)/M(IL-4) macrophage polarization highlights different activation mechanisms, e.g., time after injury. In short, in the classical macrophage polarization paradigm, naïve macrophages are pushed into the M(IFN-γ) phenotype if exposed to lipopolysaccharide (LPS) or TNF- α, while the M(IL-4) phenotype polarization is dependent on anti-inflammatory cytokines, e.g. IL-4, IL-10, IL-13 [53]. , which occurs delayed to injury. In an extensive review by Hillenbrand et al., analysis of local cytokine levels in post-SCI animal rodents showed an early (within hours) upregulation of proinflammatory cytokines IL-1β, TNFα, IL-6, and late upregulation of immunomodulatory cytokines IL-4, IL-10, IL-13 starting earliest at 24 h to 7 dpi [54].

In our experiment, we observed an Shh-associated increase in M(IL-4) polarized macrophages, characterized by CD206-expression and a decrease in M(IFN-γ) polarized macrophages, indicative through iNOS-expression. Although in vivo markers of M(IFN-γ)/M(IL-4) phenotypes are fluid and the separation effect is often difficult to determine, iNOS (M(IFN-γ)) and CD206 (M(IL-4)) are consistent, valid, widespread, and commonly recognized to differentiate M(IFN-γ) and M(IL-4) phenotypes [51, 55–57]. The observed increase of M(IL-4) polarized macrophages is typically considered anti-inflammatory and has been associated with the transition into the regenerative wound healing phase by other authors [50, 52, 53].

Our findings on the effects of intrathecally applied Shh on the integrity of the BSCB and the damping of the inflammatory response after SCI might explain the mild improvement of hindlimb locomotion observed 7 dpi. In our assessment of locomotor function, we used the grid-walk test which did not display differences between Shh and Control animals at 3 and 7 dpi, and the BBB open field score which showed significant and relevant improvements of Shh-treated animals at 7 dpi.

The BBB scoring system, appears to offer adequate sensitivity for early-stage evaluations, effectively distinguishing minor variations in the movement of hindlimbs [58, 59]. Although assessing locomotor function in our study had its challenges, our results align with prior experiments: Activation of the Shh-pathway has been previously linked to a reduction in inflammatory responses, and some studies have also directly correlated this pathway’s activation with improvements in locomotor function [10, 60, 61]. Similarly, other SCI research that focused on reducing inflammation also reported enhanced locomotor abilities in rodents [62, 63]. For instance, research by Li et al. demonstrated that mice lacking the TREM1 gene, which encodes an immune receptor that promotes inflammation, exhibited improved locomotion due to reduced inflammation [62]. These findings lend further support to the idea that managing neuroinflammation early on might be key to successful long-term recovery of locomotor function post-SCI.

Finally, several limitations need to be considered. The Gridwalk test is a recognized method for assessing coordination and weight-bearing ability following experimental SCI. However, it’s important to highlight that this test is typically conducted during later recovery phases post-injury [64]. Therefore, the subpar performance observed in the early phase evaluations of locomotor function using the Gridwalk test should have been expected. Upon reflection, we hypothesize that in the initial stages of SCI assessment, up to 7 dpi, rodents may not yet have regained sufficient coordination and muscular strength to reliably and meaningfully complete complex neurological tests with our injury model. Furthermore, we were unable to incorporate lesion size and perifocal edema volume in this study due to capacity limitations. We acknowledge that including such assessments would have enhanced our understanding of the potential effects of Shh-treatment.

## Conclusion

Topical administration of exogenous Shh to the injured spinal cord may help mitigate neuroinflammation. However, the specific effector processes remain unclear. Activation of the Shh pathway may reinforce the BSCB and potentially correlate with macrophage polarization from a pro-inflammatory M(IFN-γ) state to a regenerative M(IL-4) state during the subacute phase of SCI, which could enhance locomotor function recovery. Further research is needed to comprehensively ascertain the immediate impacts of Shh on neuroinflammation.

## Data Availability

No datasets were generated or analysed during the current study.
